# Screening tools for maternal mental health in Africa: a systematic review

**DOI:** 10.3389/fgwh.2026.1763729

**Published:** 2026-06-25

**Authors:** Joyce Jebet, Irene Mageto, Benard Mutwiri, Abednego Ongeso, Ruth Wagathu, Rose Maina, Denis Munene, Rosemary Kithuci, Peter Gatiti

**Affiliations:** 1 School of Nursing and Midwifery, Aga Khan University, Nairobi, Kenya; 2 Library Department, Aga Khan University, Nairobi, Kenya

**Keywords:** maternal mental health, postpartum, pregnancy, screening tools, systematic review

## Abstract

**Background:**

Maternal health during pregnancy and the postnatal period encompasses mental health, which is a key aspect in enhancing a positive outcome. Despite screening for other possible complications during pregnancy and the postnatal period, very little attention has been paid to screening for mental health. Screening is a key tool in early diagnosis and, eventually, timely diagnosis and management in cases where complications arise.

**Aim:**

This study aimed to assess the available maternal mental health screening tools and compare the outcome on their utilization during pregnancy and postnatal periods in the African continent.

**Methodology:**

This was a systematic review including articles published in Africa. It was registered on PROSPERO, ID CRD42024586869. The search databases were AJOL/PubMed/CINAHL, and studies published in English between 01/01/2014 and 31/12/2024 were included. Grey literature was included. The MESH terms were Maternal mental illness OR Perinatal Mental illness OR Depression OR Anxiety OR Pregnant OR postnatal AND screening tools OR Edinburgh Postnatal Depression Scale (EPDS) OR Patient Health Questionnaire (PHQ-9), OR Generalized Anxiety Disorder 7-item scale (GAD-7). To avoid risk of bias, the Cochrane risk of bias tool was utilized.

**Results:**

Most studies were cross-sectional (*n* = 15) or longitudinal cohort (*n* = 6), with sample sizes ranging from 37 to 3,311 participants. The commonly used maternal mental health screening tool was the Edinburgh Postnatal Depression Scale (EPDS). Patient Health Questionnaire (PHQ-9) was common in Ethiopia, and the Self-Reporting Questionnaire (SRQ-20) was popular in West African countries. Studies were conducted predominantly in South Africa (*n* = 12), Ethiopia (*n* = 5), Nigeria (*n* = 5), and other Sub-Saharan African countries, including Uganda, Rwanda, Kenya, Cameroon, The Gambia, and Mali.

**Conclusion:**

Maternal mental health disorders remain highly prevalent across African settings yet are under-detected. The EPDS and SRQ-20 are the most validated and widely applied screening tools; however, gaps persist in service integration, provider training, and culturally sensitive screening. Embedding screening within routine maternal and child health care could bridge existing detection and treatment gaps.

**Systematic Review Registration:**

https://www.crd.york.ac.uk/PROSPERO/view/CRD42024586869, CRD42024586869.

## 1 Introduction

This study highlights the maternal mental health screening tools that have been utilized during pregnancy and the postnatal period in Africa. A systematic review was performed for studies published between 1st January 2014 to 31st December 2024. This study was registered on PROSPERO, ID CRD42024586869.

### 1.1 Background

Pregnancy is a period that brings physical, physiological and emotional changes. Maternal mental health is a key aspect of concern during pregnancy and the postnatal period owing to the emotional changes that take place during pregnancy, which not only affect them but their families too. Maternal mental illnesses including depression, suicidal ideation and puerperal psychosis have a great impact on the mother, her family and the development of the child (1). Globally, it is estimated that 10% of pregnant women and 13% of those who have given birth suffer depression, with higher prevalence reported in the developing countries, 15.6% and 19.8% respectively (2). In low-income countries, it is estimated that one in every four pregnant women and one in every five postpartum suffer mental health problems (1). In Africa, the prevalence of depression is high, at 11.3% during pregnancy and 18.3%–18.6% postpartum (3, 4). The prevalence of maternal depression in Kenya is estimated at 30.7% (5). Another study in Kenya reports a prevalence of 7% of pregnant women and 13% of mothers (6). Depression has serious effects on both the mother and baby as the mother is not able to eat of sleep well, thus affecting the maternal—infant bonding, breastfeeding and general care of the baby (2). Suicide contributes to 20% of postpartum deaths (7). These deaths have a profound effect on the baby and the family. Children born to women with perinatal depression and anxiety have increased risk of hospitalization (8), poor cognitive, language and motor development, poor academic performance and behavioural problems (9–12).

Although all pregnant and postpartum women are predisposed to mental health disorders, poverty, extreme stress, gender and intimate partner violence, lack of social support network, biological factors, emergency and conflict situations and natural disasters contribute to the development of maternal mental health illnesses (2, 7). In addition, childbirth experiences, fetal or neonatal loss, congenital abnormalities, unplanned pregnancy, complication of pregnancy and low self-esteem contribute immensely to maternal mental health disorders (11). Women have received limited mental health attention during pregnancy or the postnatal period since there is a perception that they are protected by the hormones (12, 14, 15).

Screening is a strategy used to detect mental illness among a large population. Screening has potential benefits including early diagnosis, identifying undiagnosed cases and prevent distressing consequences (8). Ideally during pregnancy, mental health screening should be done at least once at the end of the first trimester or the beginning of the second trimester. Postnatally, this should be done at least once during the first 6 weeks after birth, which is the period in which physiological recovery of maternal reproductive organs revolute back to the non-pregnant state. In many cases, screening of mental health illnesses is based on history and physical examination (1). This may not adequately reveal the problem, especially if it is mild, or if the client is unaware of its existence. In addition, detailed history may not be taken as well as a mental status assessment may not be undertaken in most settings. Midwives and other medical staff may not be specialized or lack adequate training to carry out detailed mental health screening and manage mental health illnesses (1, 7). At times, they are very busy and often feel pressured to complete assigned tasks. Other factors that lead to lack of utilization of maternal mental health screening tools are lack of mental health resources and stigma associated with mental health (7). The common maternal mental health screening tools for maternal depression include the Edinburg postnatal depression scale (EPDS) (13, 14) and Patient Health Questionnaire (PHQ-9) tool. For anxiety, the common tool used is the Generalized Anxiety Disorder 7-item scale. Utilization of maternal mental health screening tools will help in early identification of mental illnesses, thus early treatment and prevention of serious complications, ultimately contributing to the achievement of sustainable development goal (SDG) three on health for all. The aim of this study was to determine the available tools for screening mental health among perinatal women in Africa.

## 2 Methodology

This review was conducted following the Preferred Reporting Items for Systematic Reviews and Meta-Analyses (PRISMA) guidelines to determine the existence and utilization of the screening tools.

### 2.1 Review question

Is use of standardized screening tools for maternal mental illnesses more accurate compared to no screening or usual care, among peripartum women (pregnant or postpartum) in leading to improved detection and treatment of maternal mental illnesses and better maternal and child health outcomes?

*Types of study to be included/Excluded.*

*Inclusion Criteria*:

- Maternal mental health OR perinatal mental health OR perinatal mental illness
- Screening tools OR assessment tools OR instrument
- Africa
- Published in English Language
- Published between 2014 and 2024

*Exclusion Criteria*:

- Systematic reviews
- Scoping reviews
- Meta-Analysis
- Letters to the editor
- Conference Proceedings

### 2.2 Condition or domain being studied

Maternal mental health screening tools.

*Population:* Pregnant women or postpartum women in Africa.

*Intervention:* Screening for maternal mental illnesses [e.g., using standardized screening tools like the Edinburgh Postnatal Depression Scale (EPDS), Patient Health Questionnaire (PHQ-9), or Generalized Anxiety Disorder 7-item scale (GAD-7)].

*Comparison:* No screening or usual care without standardized screening tools.

*Outcome:* Improved detection of maternal mental illnesses (e.g., detection of postpartum depression, anxiety, timely intervention and treatment, improved maternal outcomes included reduced cases of maternal mental health illnesses, child health outcomes include live births, normal growth curves, reduced cases of prematurity, low birth weight.

### 2.3 Context

African continent.

### 2.4 Data extraction (selection and coding)

After indexing the hits (with doubles removed) from the PubMed/AJOL search, two reviewers selected articles which were relevant to the research question in their title/abstract. Discrepancies were resolved through a third researcher (the PI) where necessary. Afterwards, the full text.pdfs of the selected articles were saved on a web server.

### 2.5 Eligibility criteria

The main population of interest in this study was ensuring a comprehensive and representative evidence search, this review integrated results from different research designs. These included randomized controlled trials (RCTs), case-control studies, cohorts, cross-sectional, observational studies, pre and post studies, quasi-experimental studies, and qualitative studies.

The primary studies of interest were those that utilized maternal mental health screening tools. The primary outcomes included the tools used for mental health screening during the perinatal period, diagnosis of mental illness and interventions. The secondary outcomes included maternal mental health complications and neonatal complications.

### 2.6 Search strategy

We wrote a descriptive review on the methods used for screening mental health among perinatal women. This was compared to those who have not had any form of screening and the outcome. Due to the heterogeneity of the data, we did not expect to be able to perform a meta-analysis.

We structured and conducted an electronic literature search on maternal mental health screening tools. The relevant databases searched included AJOL, PubMed, CINAHL and Scopus. First, we conducted a search using keywords on maternal mental health on the relevant databases to enhance comprehension of the existing literature on the subject matter. Some of the keywords for the preliminary searches were maternal mental health, perinatal mental health and screening tools. The preliminary search provided broader knowledge and understanding of whether there was an ongoing review on this topic.

Regarding the grey literature publications search, we examined the WHO and Ministry of Health—Kenya guidelines, and other government publications and conference proceedings informing the current review. Seemingly, the search databases and grey literature publications were purposively selected based on their relevance to availing materials on screening tools on maternal mental health. However, we limited the search to studies published in English over the last ten (10) years (2014–2024).

### 2.7 Search (MESH) terms

The keywords for this search were both Medical Subject Heading (MESH) terms and free texts. The key MESH terms were Maternal mental illness OR Perinatal Mental illness OR Depression OR Anxiety OR Pregnant OR postnatal AND screening tools OR Edinburgh Postnatal Depression Scale (EPDS) OR Patient Health Questionnaire (PHQ-9), OR Generalized Anxiety Disorder 7-item scale (GAD-7). The free texts used in our search were maternal mental health, screening and perinatal period. The detailed results from our search are presented in Table 1.

**Table 1:** Databases and search query.

Database	Search query	Limits	Results
PubMed	((“screening tools” OR “assessment tools” OR “instrument” OR “screening”) AND (((maternal mental health AND ((y_10[Filter]) AND (english[Filter]))) OR (perinatal mental health AND ((y_10[Filter]) AND (english[Filter])))) OR (perinatal mental illness AND ((y_10[Filter]) AND (english[Filter]))) AND ((y_10[Filter]) AND (english[Filter])))) AND (“Africa”[Mesh])	*Year 2014–2024 Language; English*	136
CINAHL	(“screening tool*” OR “assessment tool*” OR instrument* OR screening) AND (“maternal mental health” OR “perinatal mental health” OR “perinatal mental illness”) AND (MH “Africa+” OR Africa*)	*Year 2014–2024 Language; English*	18
SCOPUS	(TITLE-ABS-KEY (maternal AND mental AND health) OR TITLE-ABS-KEY (perinatal AND mental AND health) OR TITLE-ABS-KEY (perinatal AND mental AND illness) AND TITLE-ABS-KEY (“screening tools” OR “assessment tools” OR “instrument”) AND TITLE-ABS-KEY (africa)) AND PUBYEAR > 2013 AND PUBYEAR < 2025 AND (LIMIT-TO (DOCTYPE, “ar”) OR LIMIT-TO (DOCTYPE, “re”)) AND (LIMIT-TO (LANGUAGE, “English”))	*Year 2014–2024 Language; English*	20
AJOL	maternal mental health AND Screening AND Africa	*Year 2014–2024 Language; English*	79
CORE	maternal mental health AND Screening AND Africa	*Year 2014–2024 Language; English*	234

### 2.8 Selection of the eligible studies

After conducting the database search, we downloaded the abstracts and titles of the selected studies and saved them into the Endnote library. We removed the duplicates, and before engaging in full-text reading, two reviewers worked independently (IM & JJ; AO & RW; RM&DM; BM & RK) to screen the titles and abstracts of the relevant articles of interest from the list of references to examine the eligibility of the studies based on the inclusion and exclusion criteria. Any emerging differences on eligible studies for inclusion in full-text screening were harmonized through consensus. However, any disagreement on the study's eligibility was resolved through consultation and mutual agreement with a third independent reviewer's (PG) opinion. The articles screening process involved: reviewing the publications' abstracts and titles to exclude the non-relevant studies and reading through the primarily selected studies to discard the non-relevant articles with a specific rationale. Only the studies that met the stipulated inclusion and exclusion criteria were included in the systematic review.

### 2.9 Data extraction and management

**Figure 1:**
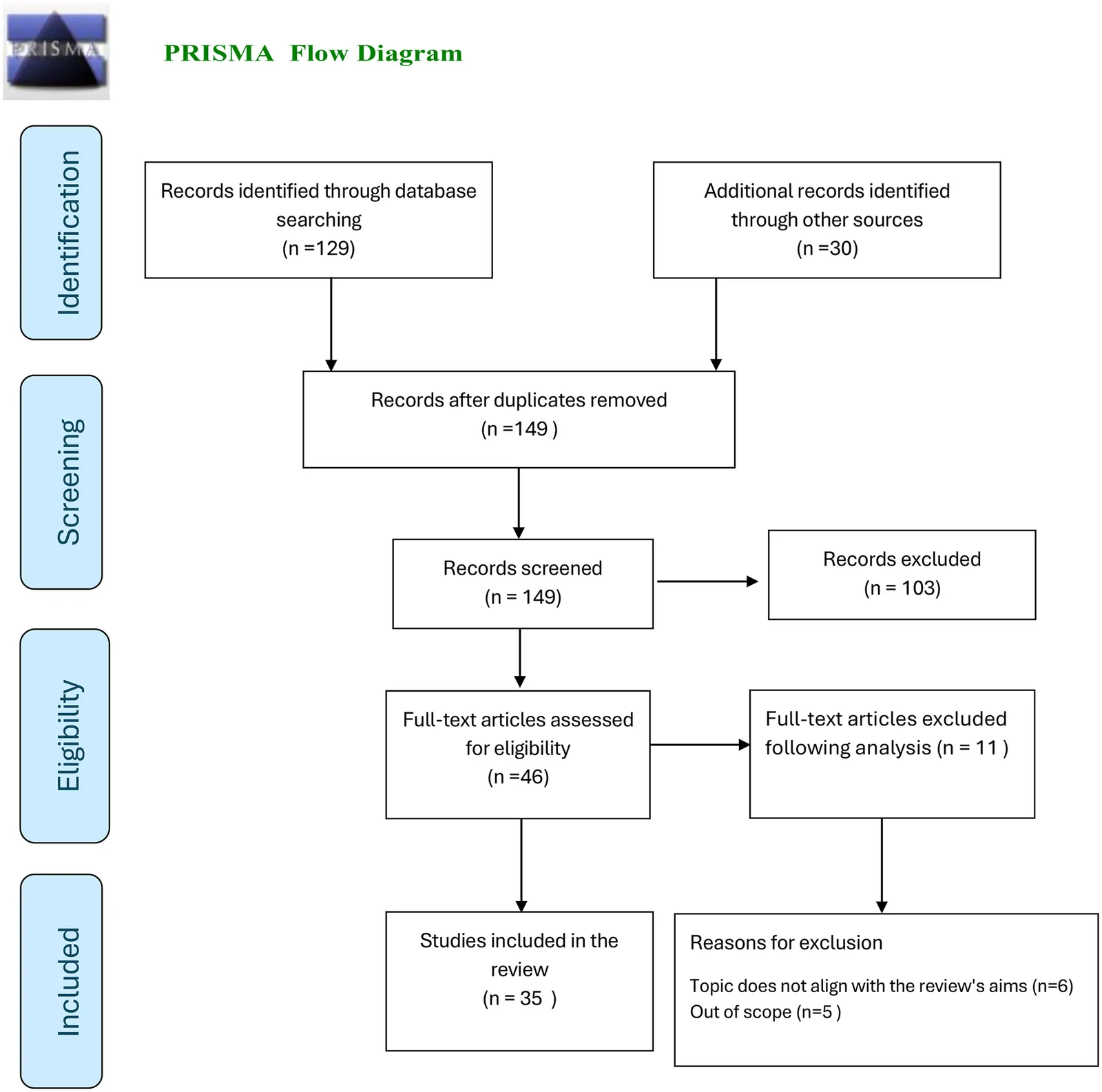
PRISMA flowchart of the search results.

We employed the PICO Framework to validate the design and approach of data extraction based on the PRISMA guidelines. A standardized tool for data extraction was adopted based on the Cochrane approach for the interventions review data collection. The independent reviewers (IM & JJ; AO & RW; RM&DM; BM) conducted data extraction using the eligible studies to be included in the final systematic review. They jointly reviewed and harmonized the extracted data through consensus and mutual engagement of the third reviewer (PG). Some of the parameters of interest in the data extraction were Author(s) and year of publication, country, study design and sample size, description of the intervention, results, and the underlying recommendations. The independent reviewers (IM& JJ; AO & RW; RM&DM; BM) conducted the data extraction and resolved variances in their final extracts before presenting (Figure 1).

### 2.10 Quality assessment (risk of bias) assessment of the included studies

To avoid the risk of bias, all relevant databases were searched. For each study, the methodological quality was assessed. Each article that had been selected for full text review was assessed for risk of bias to avoid under or over-estimation of the true intervention effect. The quality assessment of the included studies was conducted using the Grading of Recommendations Assessment, Development and Evaluation (GRADE) methodology. The double-checking of the quality of the included studies through an independent reviewer and Critical Appraisal Skills Programme (CASP) appraisal tools helped the reviewers identify both weak and robust evidence and enhance the internal validity of the final results. Some of the questions asked included the inclusion/exclusion criteria used, evidence of substantial effort to search available literature and whether the quality of the included studies was sufficiently assessed.

We assessed risk of bias in the non-randomised studies using tools appropriate to each study design. The inclusion of non-quantitative study designs was justified by the complex and multifaceted nature of maternal mental health screening and service delivery. Maternal mental health is shaped not only by measurable clinical outcomes but also by women's lived experiences, provider perspectives, stigma, cultural perceptions of mental illness, and structural barriers within health systems. Thus, qualitative studies highlight why screening interventions succeed or fail and also shed light on the acceptability and feasibility in real-world settings. For cohort and case-control studies we used the Newcastle–Ottawa Scale (NOS) (selection, comparability, outcome/exposure), and for cross-sectional studies we used an adapted NOS checklist that retained core domains (representativeness of the sample, selection, measurement, confounding, and statistical reporting). For non-randomised intervention studies (where present), we applied ROBINS-I (Risk Of Bias In Non-randomized Studies—of Interventions) to assess bias across domains relevant to interventions (pre-intervention confounding, participant selection, classification of interventions, deviations from intended interventions, missing data, measurement of outcomes, and selective reporting).

For qualitative components embedded in mixed-methods studies we appraised methodological quality using the CASP qualitative checklist. Two reviewers independently assessed each study; disagreements were resolved by discussion and, where required, by consultation with a third reviewer.

The methodological quality of the included studies was generally acceptable, with 18 studies being rated as having a low risk of bias, while 17 were classified as having a moderate risk of bias. None of the studies were classified as high risk of bias. Cross-sectional studies frequently demonstrate moderate risk due to limitations related to sampling methods, whereas most cohort studies and randomized controlled trials demonstrated lower risk of bias due to stronger methodological rigor as shown on Table 2.

**Table 2:** Appraisal tools used for risk of bias assessment.

No.	Study (Author, Year)	Study design	Appraisal tool used	Overall risk of bias
1	Baron et al. (13)	Cross-sectional	Joanna Briggs Institute (JBI) Critical Appraisal Checklist for Analytical Cross-Sectional Studies	Moderate
2	Gordon et al. (14)	Longitudinal cohort	Newcastle–Ottawa Scale (NOS)	Low
3	Kumar et al. (43)	Cross-sectional	JBI	Moderate
4	Alao et al. (33)	Cross-sectional	JBI	Moderate
5	Nwafor et al. (44)	Cross-sectional	JBI	Moderate
6	Rencken et al. (15)	Observational follow-up	NOS	Moderate
7	Rochat et al. (16)	Longitudinal cohort	NOS	Low
8	Drysdale et al. (17)	Randomized controlled trial	Cochrane Risk of Bias Tool 2	Low
9	Silverman et al. (34)	Longitudinal cohort	NOS	Moderate
10	Abrahams et al. (45)	Qualitative	Critical Appraisal Skills Programme Qualitative Checklist	Low
11	Heyningen et al. (37)	Diagnostic comparison	Quality Assessment of Diagnostic Accuracy Studies	Low
12	Sanfilippo et al. (35)	Cross-sectional comparison	JBI	Moderate
13	Ayinde et al. (18)	Mixed methods	Mixed Methods Appraisal Tool	Moderate
14	Lasater et al. (19)	Validation mixed methods	Quality Assessment of Diagnostic Accuracy Studies-2 (QUADAS-2)	Low
15	Målqvist et al. (20)	Cross-sectional	JBI	Low
16	Baumgartner et al. (36)	Cross-sectional survey	JBI	Moderate
17	Bitew et al. (31)	Qualitative	CASP	Low
18	Stewart et al. (21)	Randomized controlled trial	RoB 2	Low
19	Azale et al. (32)	Cross-sectional	JBI	Moderate
20	Abrahams et al. (22)	Validation mixed methods	QUADAS-2	Low
21	Marsay et al. (23)	Validation study	QUADAS-2	Low
22	Umuziga et al. (24)	Cross-sectional	JBI	Moderate
23	Davies et al. (38)	Validation study	QUADAS-2	Low
24	Heyningen et al. (50)	Diagnostic cross-sectional	QUADAS-2	Low
25	Redinger et al. (25)	Prospective cohort	NOS	Low
26	Bitew et al. (29) (complications)	Prospective cohort	NOS	Low
27	Bitew et al. (30) (service utilization)	Prospective cohort	NOS	Low
28	Umuziga et al. (46)	Cross-sectional	JBI	Moderate
29	Field et al. (40)	Mixed methods	Mixed Methods Appraisal Tool (MMAT)	Moderate
30	Bliznashka et al. (39)	Cluster randomized trial	Risk-of-Bias tool for randomized trials (RoB 2) RoB 2	Low
31	Comrie-Thomson et al. (26)	Cluster randomized trial	RoB 2	Low
32	Izuka et al. (43)	Cross-sectional	JBI	Moderate
33	Atuhaire et al. (41)	Cross-sectional	JBI	Moderate
34	Djatche Miafo et al. (27)	Validation cross-sectional	(QUADAS-2)	Low
35	Mukasa et al. (28)	Validation cross-sectional	QUADAS-2	Low

### 2.11 Strategy for data synthesis

The review provided a narrative summary of the maternal mental health screening tools. Besides, a tabular presentation of the included studies was made detailing the screening tools, study type, location, and outcomes. Additionally, we developed a narrative synthesis of the evident interventions and their impacts on maternal mental health.

## 3 Results

### 3.1 Study selection

The comprehensive bibliographic database search yielded 159 articles, and the deduplication process realised 149 articles, which were screened for eligibility and inclusion into the final synthesis. On initial screening, 103 articles were excluded, leaving 46 articles for full text review. The reasons for the exclusion of the 103 articles were: some studies did not utilize any maternal mental health screening tool; studies were systematic or narrative reviews, and others did not specifically focus on perinatal women. The reviewers outlined the specific reasons at each stage of the screening process. The total studies reviewed were 35.

### 3.2 Characteristics of the included studies

Table 3 summarizes the key characteristics of the thirty-five (35) studies included in this review.

**Table 3:** Study characteristics, screening tools and study outcomes.

•	Study authors/year	Study title	Country of study setting	Aim of the study	Study design	Sample size	Population	Standardized screening tools used	Purpose of using the tool	Outcomes
1.	Baron et al. (13)	Patterns of use of a maternal mental health service in a low-resource antenatal setting in South Africa	South Africa	To investigate pregnant women's patterns of use of a counselling service at a primary level obstetric facility in Cape Town, South Africa, between January 2010 and December 2011. It investigates whether these are associated with demographics, severity and risk of depressive symptoms.	Cross-sectional study	3,311 pregnant women	Pregnant women in a primary level obstetric facility in Cape Town, South Africa	Edinburgh Postnatal Depression Scale (EPDS)	To investigate the patterns of use of a mental health service in antenatal care	57.9% attended counselling; younger women were less likely to access counselling
2.	Gordon et al. (14)	Maternal depressed mood and child development over the first 5 years of life in South Africa	South Africa	To examine the long-term effects of maternal depressed mood on child development over 5 years	Multilevel regression models	1,238 pregnant women	Pregnant women and their children from peri-urban townships in South Africa	Edinburgh Postnatal Depression Scale (EPDS)	To examine the long-term effects of maternal depressed mood on child development over 5 years	Maternal depressed mood associated with lower child weight, higher aggression, and more hospitalizations; cognitive and social development unaffected
3.	Kumar et al. (43)	Mechanisms associated with maternal adverse childhood experiences on offspring's mental health in Nairobi informal settlements: a mediational model testing approach	Kenya	To examine patterns of ACEs and understanding the role of ACEs on adulthood health (i.e., physical, mental health, experience of underage pregnancy) and offspring's mental health in Kenya.	Cross-sectional study	394 mothers	Mothers from informal settlements in Kariobangi and Kangemi, Nairobi	ACE-IQ, Kessler Psychological Distress Scale (K10), Overall Health and Quality of Life items, Child Behavior Checklist	To examine the impact of maternal ACEs on adulthood health and offspring's mental health in LMIC contexts	Maternal ACEs are strong predictors of offspring mental health problems; interventions need to address maternal trauma and health to improve child outcomes
4.	Alao et al. (33)	Factors associated with common mental disorders among breastfeeding mothers in tertiary hospital nurseries in Nigeria	Nigeria	To assess the prevalence and risk factors associated with common mental disorders (CMDs) among breastfeeding mothers whose infants were admitted to Nigerian tertiary care facilities.	National cross-sectional study	895 breastfeeding mothers	Breastfeeding mothers with hospitalized infants in tertiary hospitals across six zones	WHO SRQ-20	To assess mental health and identify prevalence and risk factors of CMDs	CMD prevalence 24%; associated with education, breastfeeding support, polygamy, and prior mental health issues
5.	Nwafor et al. (44)	Prevalence and predictors of depression, anxiety, and stress symptoms among pregnant women during COVID-19-related lockdown in Abakaliki, Nigeria	Nigeria	To determine the prevalence and predictors of COVID-19-related depression, anxiety and stress symptoms among pregnant women.	Cross-sectional study	456 pregnant women	Pregnant women attending prenatal care at Abakaliki, Nigeria during COVID-19 lockdown	DASS-21	To determine the prevalence and predictors of depression, anxiety, and stress	Severe and extremely severe depression, anxiety, and stress observed; predictors include age, parity, occupation
6.	Rencken et al. (15)	Maternal mental health and caregiver competence of HIV-positive and negative women caring for their singleton newborns in KwaZulu-Natal Province, South Africa	South Africa (KwaZulu-Natal	To describe the prevalence of PND in a sample of HIV-positive and HIV-negative mothers delivering healthy singleton infants at one obstetric unit in KwaZulu-Natal (KZN) Province, South Africa, and the subsequent factors influencing neonatal behaviour and perceptions of caregiver competence.	Observational study with follow-up at 6 weeks postpartum	132 mothers (initial); 32 mothers (6-week follow-up)	HIV-positive and HIV-negative mothers of singleton newborns	Edinburgh Postnatal Depression Scale (EPDS), Mother and Baby Scales (MABS)	Assess PND prevalence, neonatal behavior, and caregiver competence in HIV-positive/negative mothers	High PND prevalence (72% baseline, 68.8% follow-up). Self-harm thoughts reported by 44.7% (baseline), 53.1% (follow-up). Lack of caregiver confidence.
7.	Rochat et al. (16)	Psychological morbidity and parenting stress in mothers of primary school children by timing of acquisition of HIV infection: a longitudinal cohort study in rural South Africa	South Africa	To investigate the prevalence of, and factors associated with, psychological morbidity amongst mothers who were still the primary caregiver of the child	Longitudinal cohort study	1,506 mothers	HIV-positive and negative mothers in rural South Africa	PHQ-9, GAD-7, Parenting Stress Index (PSI-36)	To examine psychological morbidity and parenting stress	Depression, anxiety, and parenting stress associated with HIV status and household crime exposure
8.	Drysdale et al. (17)	Father involvement, maternal depression and child nutritional outcomes in Soweto, South Africa	South Africa (Soweto)	To assess whether father involvement during and after pregnancy increased birth weight and exclusive breastfeeding through improved maternal mental health.	Randomized controlled trial (RCT)	212 mother-baby pairs	Pregnant women and postpartum mothers focusing on father involvement	Edinburgh Postnatal Depression Scale (EPDS)	Evaluate the impact of father involvement on maternal depression and child nutritional outcomes	Father involvement reduced maternal depression but had no direct impact on exclusive breastfeeding or birth weight.
9.	Silverman et al. (34)	Postpartum Mental Health in Rural South Africa: Socioeconomic Stressors and Worsening Mental Health	South Africa (Limpopo Province)	To characterize patterns of worsening mental health during the postpartum period among women in rural areas of Limpopo Province, South Africa, and to identify correlates with household demographic factors.	Longitudinal cohort study (data collected shortly after birth and 7 months postpartum)	224 postpartum women	Postpartum women in rural areas of Limpopo	World Health Organization self-reporting questionnaire (SRQ-20)	Identify patterns of worsening postpartum mental health and associated socioeconomic factors	Worsening mental health was associated with higher SRQ-20 scores shortly after birth and food insecurity at 7 months postpartum.
10.	Abrahams et al. (45)	Facilitators and barriers to detection and treatment of depression, anxiety and experiences of domestic violence in pregnant women	South Africa	To investigate facilitators and barriers of service-providers and service-users in detecting and treating pregnant women with symptoms of CMDs and experiences of domestic violence.	Qualitative study	37 healthcare workers and 38 pregnant women	Pregnant women attending MOUs and healthcare workers	EPDS, psychological distress questionnaire, domestic violence questionnaire	To detect symptoms of CMDs and experiences of domestic violence	Identified facilitators and barriers to detection and treatment at system, provider, and patient levels
11.	Van Heyningen et al. (37)	Comparison of mental health screening tools for detecting antenatal depression and anxiety disorders in South African women	South Africa	To compare five widely-used questionnaires in a sample of pregnant women in urban South Africa [Edinburgh Postnatal Depression Scale (EPDS), the Patient Health Questionnaire (PHQ-9), the Kessler Psychological Distress scale (K10) and a shortened 6-item version (K6), the Whooley questions and the two-item Generalised Anxiety Disorder scale (GAD-2)].	Quantitative cross-sectional study	376 pregnant women	Pregnant women attending primary care antenatal clinic in urban Community Health Centre	Edinburgh Postnatal Depression Scale (EPDS)	To compare performance of five screening tools in detecting antenatal depression and anxiety disorders in low-income pregnant women in primary care setting	32% diagnosed with MDE and/or anxiety disorders
								Patient Health Questionnaire (PHQ-9)		All tools showed moderate-high performance (AUC 0.78–0.85)
								Kessler Psychological Distress scale (K10 and K6)		EPDS best for MDE detection
								Whooley questions		K10/K6 best for anxiety detection
								Two-item Generalised Anxiety Disorder scale (GAD-2)		Short tools (K10/K6) and ultra-short tool (Whooley questions) performed well and may be feasible for low-resource settings
12.	Sanfilippo et al. (35)	Expression of antenatal symptoms of common mental disorders in The Gambia and the UK: a cross-sectional comparison study	The Gambia and UK	To compare Gambian pregnant women's responses to the Edinburgh Postnatal Depression Scale (EPDS) and Self-reporting Questionnaire (SRQ-20) and (b) compare responses to the EPDS in pregnant women in The Gambia and UK.	Cross-sectional comparison study	221 pregnant women from The Gambia and 368 pregnant women from the UK	Pregnant women between 10 and 30 weeks gestation in The Gambia and pregnant women around 21 weeks gestation in the UK	Edinburgh Postnatal Depression Scale (EPDS)	To compare (a) Gambian pregnant women's responses to the EPDS and SRQ-20 and (b) compare responses to the EPDS between pregnant women in The Gambia and UK to understand cultural differences in expressing mental health symptoms	Gambian EPDS and SRQ-20 scores were moderately correlated (*r*s = 0.6, *p* < 0.001)
								Self-reporting Questionnaire (SRQ-20)		Different proportions identified with high symptoms (SRQ-20 = 42% vs. EPDS = 5% using highest cut-off)
										UK participants had higher EPDS scores (*M* = 6.5) than Gambian participants (*M* = 4.4)
										Somatic symptoms were more frequently endorsed by Gambian sample
13.	Ayinde et al. (18)	Quality of perinatal depression care in primary care setting in Nigeria	Nigeria	To assess the existing organization of service for maternal mental health, the actual care delivered for perinatal depression, as well as the quality of the care received by affected women presenting to primary care clinics in Ibadan, Nigeria.	Mixed method study	2,989 pregnant women screened	Pregnant women attending primary maternal care clinics	Edinburgh Postnatal Depression Scale (EPDS)	To assess: Organizational structure for maternal mental health service delivery	Only 31 out of 218 depressed women were identified by providers
						20 facility managers interviewed		Assessment of Chronic Illness Care (ACIC) tool	Actual care delivered for perinatal depression	All facilities had poor capacity to offer quality chronic care (low ACIC scores)
								Key informant interviews	Quality of care received by affected women in primary care clinics	Major inadequacies in organizational structure and administration
										Large treatment gap for perinatal depression
14.	Lasater et al. (19)	Reliability and validity of a perinatal depression screening instrument in rural Mali	Mali	To develop a locally valid and reliable screening instrument for use in identifying pregnant women and mothers of young children with a local depression syndrome, dusukasi, in rural Mali.	Mixed methods Cross-sectional	180 pregnant women and mothers with children under age 2	Pregnant women and mothers with children under age 2 years	Edinburgh Postnatal Depression Scale (EPDS)	To develop and validate a locally valid and reliable screening instrument for identifying pregnant women and mothers with dusukasi (local depression syndrome) in rural Mali	The adapted screening tool demonstrated construct validity for identifying dusukasi in rural Malian women (Full 28-item scale showed good internal consistency (Cronbach’s alpha = 0.92)
								Hopkins Symptom Checklist (HSCL-25)		
15.	Målqvist et al. (20)	Screening for Antepartum Depression Through Community Health Outreach in Swaziland	Swaziland	To screen women for depression during the third trimester of pregnancy	Cross-sectional study	1,038 pregnant women	Pregnant women in third trimester attending antenatal care	Edinburgh Postnatal Depression Scale (EPDS)	To measure the burden of antepartum depression and identify risk factors among women in a peri-urban community through community health outreach with “Mentor Mothers”	22.7% of women had EPDS scores ≥13
										Depression was not associated with HIV status, age or employment
										Women with multiple socioeconomic stressors were more likely to score highly on EPDS
16.	Baumgartner et al. (36)	Maternal mental health in Amhara region, Ethiopia: a cross-sectional survey	Ethiopia	To describe the mental health status of women who had given birth in the last 24 months in the Amhara region of Ethiopia.	Cross-sectional survey	1,294	Women aged 15–49 who delivered in previous 24 months	SRQ-20 (WHO Self-Reporting Questionnaire)	To screen for common mental disorders (CMD) including depression, anxiety, and somatic symptoms	32.8% had probable CMD using 4/5 cutoff 19.8% had probable CMD using 7/8 cutoff 14.5% reported suicidal thoughts
17.	Bitew et al. (31)	Stakeholder perspectives on antenatal depression and the potential for psychological intervention in rural Ethiopia: A qualitative study	Ethiopia	To understand women and healthcare workers’ (HCWs) perspectives of antenatal depression, their treatment preferences and potential acceptability and feasibility of psychological interventions in the rural Ethiopian context.	Qualitative study	23 (8 women, 8 PHC workers, 7 health extension workers)	Women who scored ≥5 on PHQ-9 during pregnancy and healthcare workers	PHQ-9	To identify women with antenatal depressive symptoms for study participation	Explored perspectives on antenatal depression and psychological interventions
18.	Stewart et al. (21)	The impact of maternal diet fortification with lipid-based nutrient supplements on postpartum depression in rural Malawi: a randomised-controlled trial	Malawi	To test the hypothesis that women taking a fatty acid-rich lipid-based nutrient supplement (LNS) would have fewer depressive symptoms postpartum than those taking iron-folate (IFA) or multiple-micronutrient (MMN) capsules.	Randomized controlled trial	1,391 women (LNS = 462, MMN = 466, IFA = 463)	Pregnant women and up to 6 months postpartum	SRQ-20 and EPDS	To screen for maternal depressive symptoms	No significant differences in depressive symptoms between groups receiving different supplements.
19.	Azale et al. (32)	Treatment gap and help-seeking for postpartum depression in a rural African setting	Ethiopia	To determine the proportion of women with PPD who sought help form a health facility and the associated factors.	Community-based cross-sectional survey	3,147 screened	Women between 1 and 12 months postpartum	PHQ-9	To screen for depressive symptoms (cutoff ≥5)	Only 4.2% accessed mental health care, 12.7% had any health service contact
20.	Abrahams et al. (22)	Validation of a brief mental health screening tool for pregnant women in a low socio-economic setting	South Africa	To use a cognitive interviewing technique to validate the content and structure of a 4-item screening tool, to adapt the tool accordingly, and to use receiver operating curve (ROC) analysis to determine the optimum cut-point for identifying pregnant women with symptoms of CMD.	Mixed methods validation study	66 pregnant women	Pregnant women at first antenatal visit	4-item screening tool (Whooley questions, Generalized Anxiety Scale (GAD-2), Edinburgh Postnatal Depression Scale (EPDS) validated against EPDS	To validate a brief mental health screening tool for pregnant women	Validation and feasibility results of screening tool
21.	Marsay et al. (23)	Validation of the Whooley questions for antenatal depression and anxiety among low-income women in urban South Africa	South Africa	To evaluate the Whooley case finding questions as a potential screening tool, against a clinical interview and the Edinburgh Postnatal Depression Scale (EPDS).	Mixed methods validation study	145 pregnant women	Pregnant women at antenatal care	Whooley questions validated against EPDS & clinical interview	To validate Whooley questions for screening depression and anxiety	The Whooley questions showed good utility as a brief screening tool for perinatal mental disorders in South Africa, with sensitivity of 73.2% and specificity of 76.4% when including the 'help' question, making it comparable to the longer EPDS screening tool and suitable for routine use in busy clinical settings.
22.	Umuziga et al. (24)	A cross-sectional study of the prevalence and factors associated with symptoms of perinatal depression and anxiety in Rwanda	Rwanda	To explore the prevalence of symptoms of perinatal depression and anxiety in Rwanda, and factors associated with them	Cross-sectional	165	Women in the perinatal period (pregnant and up to 1 year postpartum)	Edinburgh Postnatal Depression Scale (EPDS), Zung Self-rating Anxiety Scale (SAS)	To assess symptoms of perinatal depression (EPDS) and anxiety (SAS)	Among antenatal women: 37.6% had symptoms indicating possible depression (EPDS ≥10), 28.2% had symptoms of clinical anxiety (SAS >45).
										Among postnatal women: 63.6% had symptoms of possible depression, 48.1% had symptoms of probable anxiety.
23.	Davies et al. (38)	Adaptation and validation of a structured version of the Hamilton Depression Rating Scale for use by non-clinicians in South Africa (AFFIRM-HDRS)	South Africa	To adapt and validate a structured version of the HDRS to be used by non-clinicians in low-income settings	Adaptation, translation and validation study	187	isiXhosa-speaking perinatal women in Khayelitsha, Cape Town	AFFIRM-HDRS (adapted Hamilton Depression Rating Scale), Edinburgh Postnatal Depression Scale (EPDS)	To adapt and validate a structured version of the Hamilton Depression Rating Scale (HDRS) for use by non-clinicians in low-income settings	The AFFIRM-HDRS demonstrated good validity and reliability, showing significant correlations with the EPDS (Rho = 0.60 and 0.43, *p* < .001) and acceptable internal consistency (Cronbach's alpha = 0.74). It performed consistently across different raters (inter-rater reliability: 0.97–0.98) and overtime (test-retest reliability: 0.90, 95% CI: 0.86–0.93).
										The AFFIRM-HDRS is adequately structured to be used by non-clinicians in an isiXhosa speaking perinatal population.
24.	Heyningen et al. (50)	Comparison of mental health screening tools for detecting antenatal depression and anxiety disorders in South African women	South Africa	To measure the diagnostic prevalence of depression among pregnant women and identify associated psychosocial risk factors	Cross-sectional	376	Pregnant women attending their first antenatal visit	Mini-International Neuropsychiatric Interview (MINI Plus)	To measure the diagnostic prevalence of depression among pregnant women and identify associated psychosocial risk factors	22% of women had major depressive episodes (MDE); food insecurity, intimate partner violence, lack of social support, and past psychiatric history were significant risk factors
										Women who presented a high risk for suicide were referred to specialist care after screening. Women who were diagnosed with a common mental disorder such as MDE were offered counselling with the mental health officer.
25.	Redinger et al. (25)	Antenatal depression and anxiety across pregnancy in urban South Africa	South Africa	To investigate the incidence and persistence of both depression and anxiety across pregnancy; identify risk factors associated with both transient and persistent symptoms of both; and explore potentially modifiable mechanisms in the development of depression and anxiety in pregnancy.	Prospective cohort study	649	Pregnant women aged 18–44 years	Edinburgh Postnatal Depression Scale (EPDS), State-Trait Anxiety Inventory (STAI-6)	To assess depression and anxiety at two point (early and later pregnancy) and explore risk factors for transient vs. persistent symptoms	High rates of antenatal depression and anxiety were noted.
										Early depression predicted later family stress, and early family stress predicted later anxiety.
										Early identification of depression and anxiety, in the first trimester, is critical for prevention and treatment.
26.	Bitew et al. (29)	Antenatal depressive symptoms and perinatal complications: a prospective study in rural Ethiopia	Ethiopia	To investigate whether antenatal depressive symptoms predict perinatal complications in a rural Ethiopia setting	Population-based prospective study	1,240	Pregnant women in their second or third trimester	Patient Health Questionnaire (PHQ-9)	To assess the association between antenatal depressive symptoms and perinatal complications	28.7% of women had antenatal depressive symptoms
										Women with antenatal depressive symptoms had more than twice the odds of pregnancy, labor, and postpartum complications, including hemorrhage, prolonged labor, severe headache, and neonatal complications such as difficulty in breathing
27.	Bitew et al. (30)	Antenatal depressive symptoms and utilisation of delivery and postnatal care: a prospective study in rural Ethiopia	Ethiopia	To examine whether antenatal depressive symptoms are associated with use of maternal health care services.	Population-based prospective study	1,251	Pregnant women in their second or third trimester	Patient Health Questionnaire (PHQ-9)	To examine whether antenatal depressive symptoms are associated with the use of maternal healthcare services (institutional delivery and postnatal care)	High levels of antenatal depressive symptoms (PHQ score 5 or higher) were found in 28.7% participating women.
										Women with antenatal depressive symptoms had increased odds of institutional birth, but it was often unplanned due to emergencies; depressive symptoms were linked to increased assisted deliveries as compared to women without these symptoms.
28.	Umuziga et al. (46)	Antenatal depressive symptoms in Rwanda: rates, risk factors, and social support	Rwanda	To examine the relationship between antenatal depressive symptoms and social support across several relationships among women attending antenatal care services.	Descriptive cross-sectional	396	Pregnant women attending antenatal care	Edinburgh Postnatal Depression Scale (EPDS), Maternity Social Support Scale (MSSS)	To examine the relationship between antenatal depressive symptoms and social support across several relationships	Prevalence of antenatal depressive symptoms was 26.6%
										Poor partner support and unplanned pregnancy were significant predictors of depression
										Friend support was not a significant factor in multivariate analysis
29.	Field et al. (40)	Domestic and intimate partner violence among pregnant women in a low resource setting in South Africa: a facility-based, mixed methods study	South Africa	To determine associations between mental illness, demographic, psychosocial and economic factors with experience of intimate partner violence (IPV) among pregnant women in a low resource setting in Cape Town and to explore the contextual elements pertaining to domestic violence.	Facility-based mixed methods study (cross-sectional survey + qualitative case note analysis)	376 (survey) + 95 (case note analysis)	Pregnant women attending antenatal care	- Expanded Mini-International Neuropsychiatric Interview (MINI) - Revised Conflict Tactics Scale (CTS2) - Multidimensional Scale of Perceived Social Support (MSPSS) - Household Food Security Survey Module (HFSSM) - Risk Factor Assessment (RFA)	- MINI: To assess mental health status (depression, anxiety, suicidality, substance use) - CTS2: To assess intimate partner violence (IPV) - MSPSS: To assess social support - HFSSM: To assess food insecurity - RFA: To assess psychosocial risk factors	15% of women reported IPV in the past 6 months
										IPV was associated with food insecurity, unemployment, being unmarried, past abuse, and unwanted pregnancy
										IPV was strongly associated with depression, anxiety, and suicidal thoughts
										55% of women experiencing domestic violence were abused by someone other than an intimate partner (e.g., family members)
										Violence was often perceived as “normal” in the community
30.	Bliznashka et al. (39)	Effects of a community health worker delivered intervention on maternal depressive symptoms in rural Tanzania	Tanzania	To evaluate the effect of a community health worker (CHW) delivered home visit responsive stimulation, health and nutrition intervention, and conditional cash transfers (CCTs) for antenatal care and child growth monitoring attendance on maternal depressive symptoms.	Cluster-randomized controlled trial (cRCT)	593	Pregnant women and mothers with a child <12 months in 12	Hopkins Symptoms Checklist-25 (HSCL-25)	To assess maternal depressive symptoms at baseline, midline (9 months), and endline (18 months)	Community health worker (CHW) intervention significantly reduced HSCL-25 scores at 9 months and 18 months
										CHW + Conditional Cash Transfer (CCT) also reduced depressive symptoms but was not statistically significant
										No additional benefit of CCTs beyond CHW intervention
										Suggests integrated community-based interventions can improve maternal mental health
31.	Comrie-Thomson et al. (26)	Engaging women and men in the gender-synchronised community-based Mbereko + Men intervention to improve maternal mental health and perinatal care-seeking in Manicaland, Zimbabwe: A cluster-randomised controlled pragmatic trial	Zimbabwe	To test the effectiveness of the Mbere­ko + Men program on maternal mental health at 0–6 months after childbirth.	Cluster-randomized controlled trial (cRCT), two-arm parallel design	457 women & 242 men (pre-intervention), 433 women & 273 men (post-intervention)	Women who had given birth in the past 0–6 months and their male coparents	Edinburgh Postnatal Depression Scale (EPDS)	To assess maternal depressive symptoms before and after the Mbereko + Men community-based intervention	Mean EPDS scores declined by 63% in the intervention arm, compared to 45% in the control arm
										Increased antenatal/postnatal care-seeking behaviors in the intervention arm
										Improved male support and coparents’ decision-making dynamics in the intervention arm
32.	Izuka et al. (43)	Evaluation of Anxiety and Depression among Pregnant Women in Enugu, Nigeria	Nigeria	To evaluate depression and anxiety among pregnant women who receive antenatal care in four randomly selected hospitals in Enugu, Nigeria.	Cross-sectional	434	Pregnant women	Hospital Anxiety and Depression Scale (HADS)	To assess the prevalence of anxiety and depression among pregnant women and identify associated risk factors	Prevalence: 9.7% had depressive symptoms, 11.1% had borderline depression, 10.1% had anxiety symptoms, and 15.7% had borderline anxiety
										Significant factors for depression: Gestational age <28 weeks, husband's unemployment
										Significant factors for anxiety: Lower education level, husband's unemployment
										Parity was linked to borderline anxiety, with nulliparous women at higher risk
33.	Atuhaire et al. (41)	Prevalence of postpartum depression and its association with diabetes mellitus among mothers in public health facilities in Mbarara, Southwestern Uganda	Uganda	To determine the prevalence of postpartum depression and its association with diabetes mellitus among mothers in Mbarara District, southwestern Uganda.	Facility Based cross-sectional study	309	Mothers in the postpartum period	Mini-International Neuropsychiatric Interview (MINI 7.0.2)	Determining prevalence of postpartum depression and its association with diabetes mellitus among mothers in Mbarara District, southwestern Uganda.	PPD prevalence of PPD among mothers of 6th weeks to 6th months postpartum period in Mbarara was 40.5% (95% CI: 35.1–45.1) and it was statistically significantly associated with diabetes mellitus in mothers between 6 weeks and 6 months postpartum.
										The prevalence of diabetes mellitus among mothers with PPD was 28% compared to 13.6% among mothers without PPD.
										Mothers with PPD had 3 times higher odds of being newly diagnosed with diabetes as compared to those without PPD (aOR = 3.0, 95% CI: 1.62–5.74, *p* = 0.001).
34.	Djatche Miafo et al. (27)	Validation of the Edinburgh postnatal depression scale and prevalence of depression among adolescent mothers in a Cameroonian context	Cameroon	To determine the cut-off for teenage mothers in Cameroon	Cross-sectional and analytical	1,633	Adolescent mothers	Edinburgh Postnatal Depression Scale (EPDS)	To define a cut-off score or the prevalence of perinatal depression in adolescent mothers and determine the cut-off for teenage mothers in Cameroon.	The prevalence of perinatal depression was 60.8% (95% CI = 58.5, 63.2). The cut-off score for this population was ≥ 11. Sensitivity was 92.6% (95% CI = 0.913, 0.939) specificity 53.2% (95% CI = 0.508, 0.556), PPV 75.5% and NPV 80.2%.
35.	Mukasa et al. (28)	The Luganda Edinburgh Postnatal depression scale: cross-cultural adaptation and validation for prenatal screening of depression in a Ugandan sample	Uganda	To Adapt and validate the Luganda Edinburgh Postnatal Depression Scale (EPDS-L) for screening prenatal depres­sion at Kawempe National Referral hospital (KNRH)	Cross-sectional; study	100	Prenatal mothers	Edinburgh Postnatal Depression Scale (EPDS-L)	To Adapt and validate the Luganda Edinburgh Postnatal Depression Scale (EPDS-L) for screening prenatal depres­sion	EPDS-L had Cronbach's-Alpha of 0.8515. At cut-off of 13, sensitivity was 62.86%, specificity-100%, PPV-100% and NPV-83.3%. AUC was 0.99. Performance was better at cut-off of 10, with sensitivity-97.14% and specificity-98.46%.
										The EPDS-L is reliable at cut-off of 13 but performs even better at cut-off of 10.

Most studies utilized standardized screening instruments such as Edinburgh Postnatal Depression Scale (EPDS) which was most widely used (15–30). Patient Health Questionnaire (PHQ-9) was common in Ethiopia (31–34). The Self-Reporting Questionnaire (SRQ-20) were popular in West African countries (35–38). The Whooley questions (24, 25, 39), GAD-2 (24, 39), and Kessler scales (K10/K6) was used for anxiety/depression detection.

Validation studies were conducted to adapt tools culturally and linguistically (30, 40), emphasizing sensitivity, specificity, and feasibility for use by non-specialists in low-resource settings. Sample sizes varied greatly with small qualitative studies (*n* ≈ 23–100) like (33) and large surveys having more than 1,000 respondents (15, 34). Most studies recruited women from antenatal/postnatal clinics or community health centers, indicating reliance on facility-based screening, though some used community outreach (22, 41), highlighting potential for broader, decentralised screening strategies. Overall, methodological diversity enhanced the robustness of findings yet also pointed to gaps in longitudinal monitoring and intervention-based research, especially in settings outside South and East Africa.

### 3.3 Characteristics of participants in the included studies

**Figure 2:**
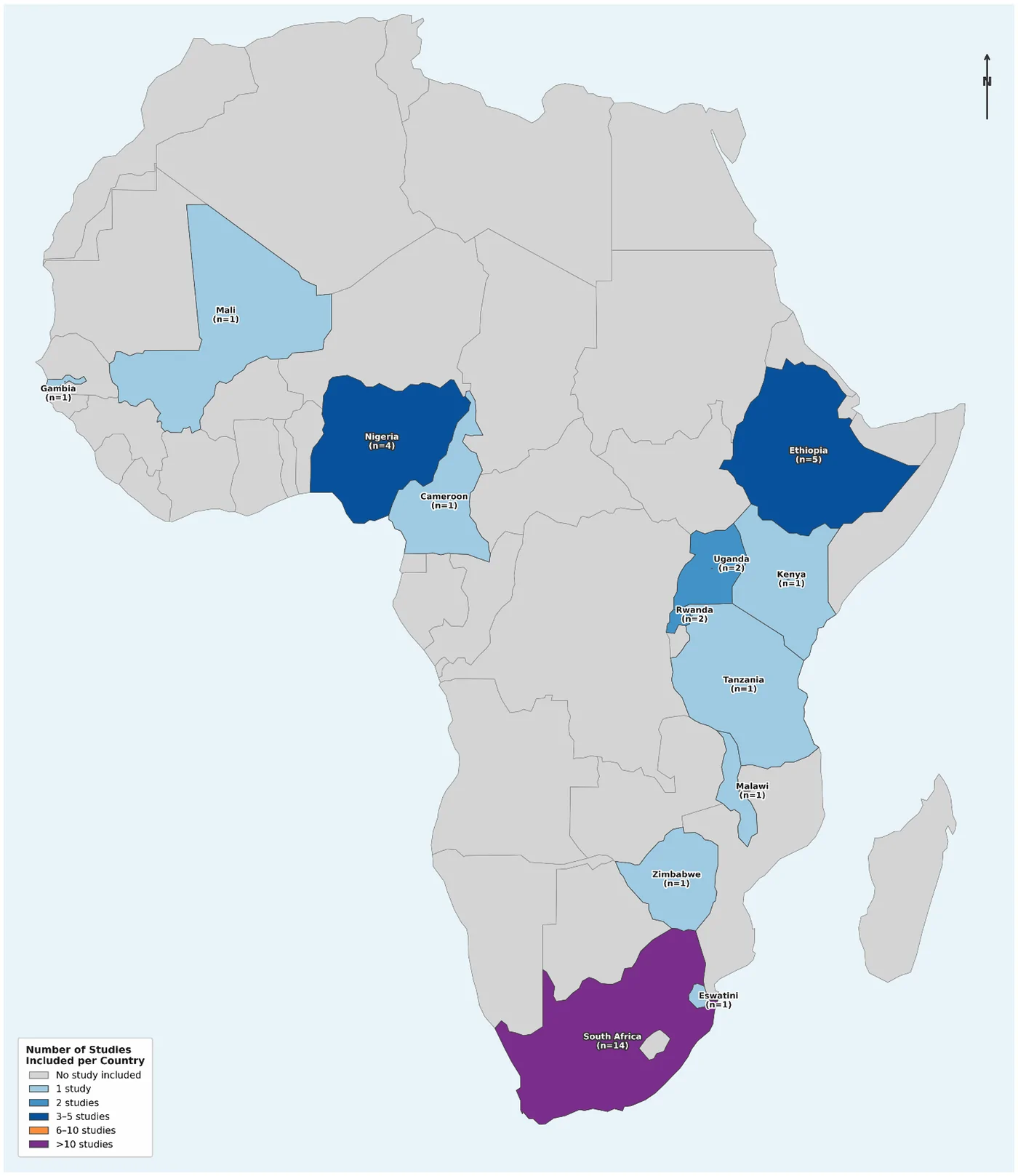
Map showing countries represented in the maternal mental health screening study.

The participants across the studies primarily comprised pregnant and postpartum women in diverse African settings, including urban, peri-urban, and rural communities (Figure 2). Most participants were women aged between 15 and 49 years, with a few studies specifically targeting subgroups such as adolescent mothers (29) and HIV-positive women (17, 18).

Socioeconomic vulnerability was a recurring characteristic. Many women lived in low-resource settings with high rates of poverty, unemployment, food insecurity, and low educational attainment (27, 37). Studies from South Africa (39, 42) and Ethiopia (34) reported that intimate partner violence (IPV), unplanned pregnancies, and limited social support were prevalent among the study populations.

Health-related factors such as HIV status (17), diabetes mellitus (43), and history of adverse childhood experiences (ACEs) (44) were also commonly explored, suggesting a focus on biopsychosocial determinants of maternal mental health. The participants' mental health status varied, with reported prevalence of depressive symptoms ranging from 9.7% (45) to 72% (17), depending on the tool used and the setting. These findings emphasize the intersectionality of demographic, socioeconomic, and clinical characteristics, underscoring the importance of culturally adapted and contextually appropriate screening strategies (28, 41).

### 3.4 Methods of the reviewed studies

The studies employed a range of methodologies, primarily quantitative cross-sectional designs (15, 22, 29, 30, 33, 34, 38, 44–46), longitudinal cohort studies, which followed women through pregnancy to the postnatal period (16, 18, 27, 31, 32, 36), and randomized controlled trials (RCTs) (23, 28). A subset included qualitative (33, 47) and mixed methods designs (20, 21, 25, 42).

### 3.5 Synthesis of maternal mental screening tools

The commonly used maternal mental health screening tool was the Edinburgh Postnatal Depression Scale (EPDS), which was utilized in twenty out of thirty five reviewed studies (15, 17, 18, 20, 21, 23–25, 27, 29, 34, 36, 38, 39, 41, 42, 44–46). Studies comparing the screening tools highlight the strong performance of the EPDS (39). Additional studies in Rwanda identified substantial antenatal and postnatal depressive symptoms—up to 63.6% postpartum—and linked these to factors such as poor partner support and unplanned pregnancy (26, 48). In Swaziland (22), reported that 22.7% of women scored ≥13, with socioeconomic stressors increasing risk. In Mali, the EPDS and HSCL were used to develop a culturally grounded tool for *dusukasi*, identifying nearly half of women with depression or anxiety (21). Some other studies (11) used the patient health questionnaire-9 (PHQ-9) (16, 19, 24, 26, 28, 30, 32, 33, 35, 43, 48). The Mini International Neuropsychiatric Interview (MINI) was used by four studies alongside other tools (18), used alongside EPDS) and (33) used alongside PHQ-9; (32). Other screening tools used were Generalized Anxiety Disorder-7 (GAD-7) (44) and Self-Reporting Questionnaire-20 (SRQ-20) used by (22).

### 3.6 Outcomes

Prevalence of maternal mental disorders was high across multiple African settings. Antenatal and postnatal depression rates ranged from 10% to 43%, with variability linked to geographic and population differences (15, 16, 22, 32). Anxiety disorders were also common, with prevalence estimates between 15% and 30% (27, 36). Risk factors for maternal mental illness identified included low socioeconomic status, food insecurity, HIV infection, intimate partner violence, and lack of social support (20, 27, 36). Protective factors such as partner involvement, strong social networks, and household economic stability were associated with reduced risk of the illnesses (28). Consequences of poor maternal mental health were reported for both mothers and infants. Adverse outcomes included low birthweight, preterm delivery, poor breastfeeding practices, and impaired infant growth and development (18, 23, 38). Validation studies of screening tools such as the Edinburgh Postnatal Depression Scale (EPDS), Patient Health Questionnaire-9 (PHQ-9), Self-Reporting Questionnaire-20 (SRQ-20), and Hopkins Symptom Checklist (HSCL-25) demonstrated acceptable reliability and validity across diverse African populations (24–26). One study recommended locally adapted cut-off points to improve diagnostic accuracy (25).

Intervention outcomes showed that community-based and psychosocial interventions reduced maternal depression and anxiety symptoms. Task-sharing models using community health workers and male involvement strategies were effective in improving maternal mental health and strengthening family relationships (19, 28, 41). Integrated psychosocial and nutritional interventions also improved both maternal mental health and infant feeding outcomes (23). Health system–related outcomes demonstrated the feasibility of integrating maternal mental health screening into routine antenatal and postnatal care. However, common barriers included staff shortages, stigma, and limited referral systems (22, 24, 40, 49). Studies conducted during the COVID-19 pandemic reported elevated prevalence of depression, anxiety, and stress symptoms among perinatal women, attributed to social restrictions, economic challenges, and reduced access to health services (45, 46).

## 4 Discussion

This systematic review aimed to assess the available maternal mental health screening tools and compare the outcome on their utilization during pregnancy and postnatal periods in the African continent. It synthesized evidence from thirty-five studies that were conducted in African countries. The studies included a range of research designs ranging from cross-sectional surveys, longitudinal cohorts, randomized controlled trials, mixed methods studies, and validation studies.

The findings of this systematic review show that the study participants were predominantly pregnant and postpartum women drawn from a wide range of African settings, including urban, peri-urban, and rural communities (22, 29, 30, 45, 47, 48). Most of the women were aged between 15 and 49 years, reflecting the typical reproductive age group. Some studies, however focused on particularly vulnerable subpopulations such as adolescent mothers, diabetic and HIV-positive women (17, 29, 43). These groups face unique developmental, social, and clinical challenges that heighten their risk of poor perinatal mental health outcomes.

Across the studies, socioeconomic vulnerability emerged as a consistent and defining characteristic. Many women lived in low-resource environments marked by high levels of poverty, unemployment, food insecurity, and low educational attainment (33, 35, 45, 46). This structural disadvantage was compounded by interpersonal challenges such as intimate partner violence, unplanned pregnancies, and limited social support, as reported in studies from South Africa, Ethiopia, and other regions. These conditions create multiple and overlapping stressors that significantly influence women's psychological wellbeing during pregnancy and after childbirth.

Health-related factors also contributed to the prevalence of perinatal mental disorders. Studies commonly examined the impact of chronic illnesses such as HIV and diabetes mellitus, as well as the long-term effects of adverse childhood experiences (17–19, 43). This points to a growing recognition of the biopsychosocial determinants of maternal mental health, where biological vulnerability, psychological histories, and social contexts interact to shape outcomes. Unsurprisingly, the prevalence of depressive symptoms varied markedly between studies, ranging from under 10% to over 70%. Differences in the study settings, sample characteristics, and screening tools used likely contribute to this wide variability. These disparities highlight the importance of contextually appropriate, culturally adapted approaches to mental health screening and assessment.

The Edinburgh Postnatal Depression Scale (EPDS) was the most utilized screening tool across sixteen studies in South Africa, Rwanda, Swaziland, Mali, Malawi, and Cameroon, while other tools showed effectiveness in specific cultural and linguistic contexts. The EPDS is possibly adapted owing to its simplicity in use and local adaptability. Although other tools have been used, they are not commonly used in the African setting. The predominance of the EPDS across the studies highlights its acceptability and adaptability in diverse African populations. Several studies not only used the EPDS to screen for depressive symptoms but also validated or compared it with other tools to determine optimal screening approaches in resource-constrained health systems.

The most prevalent common mental disorders (CMDs) were depression, anxiety, and stress while antenatal or postnatal depression ranged from 20% to over 60%, with particularly high rates observed among adolescent mothers in Cameroon (29) and mothers of hospitalized infants in Nigeria (35). The high prevalence of depression is linked to stressors such as food insecurity, stigma, intimate partner violence, low social support and parental stress (16–19). Some studies validated brief screening tools (24, 25), showing good utility as brief screening tools comparable to longer instruments.

Methodologically, the studies reviewed employed a mix of quantitative and qualitative research designs. The majority used cross-sectional methods, which are valuable for estimating prevalence and identifying associated factors but offer limited insight into temporal relationships or changes over time. A smaller number of longitudinal cohort studies (18, 31) followed women through pregnancy and into the postnatal period, providing deeper understanding of how symptoms evolve and how risk factors exert their influence over time. Randomized controlled trials (23, 28) were fewer but provided important evidence on the effectiveness of tailored interventions for improving maternal mental health.

The studies demonstrate that cut-off scores for screening tools vary across contexts and populations, reflecting differences in cultural expression of psychological distress and the purpose of screening. For the EPDS, cut-off scores commonly range between 10 and ≥13, depending on the study setting and population. For example, research in Rwanda and Swaziland used EPDS cut-offs around ≥10 or ≥13 to identify probable depression, while validation studies in Cameroon identified an optimal cut-off of ≥11 among adolescent mothers (29). Similarly, adaptation studies in Uganda suggested that a cut-off of 10 improved sensitivity (97.1%) and specificity (98.5%) (30), compared with the conventional cut-off of 13. Other tools also demonstrated variable thresholds; the PHQ-9 frequently used a cut-off of ≥5 for identifying depressive symptoms in Ethiopian community studies, while the SRQ-20 applied thresholds between 4/5 and 7/8 to identify probable common mental disorders. These variations underscore the importance of context-specific validation of screening thresholds before routine implementation in maternal health services.

Several studies specifically examined the psychometric performance of screening tools, demonstrating generally good validity and reliability in African perinatal populations (15, 29). The EPDS consistently showed strong performance for detecting depressive symptoms and major depressive episodes. Comparative analyses in South Africa reported moderate to high diagnostic accuracy (AUC 0.78–0.85) when EPDS was compared with other instruments for detecting major depressive episodes and anxiety disorders (36). Similarly, adapted versions of screening instruments demonstrated high internal consistency and reliability. For instance, validation studies in Mali reported a Cronbach's alpha of 0.92 for an adapted depression scale (21), while the Luganda version of EPDS demonstrated Cronbach's alpha of 0.85, indicating strong reliability (30) [NO_PRINTED_FORM] The adapted AFFIRM-HDRS also showed high inter-rater reliability (0.97–0.98) and test–retest reliability (0.90), suggesting that structured diagnostic tools can be administered reliably by trained non-clinicians.

Qualitative and mixed-methods studies enriched the evidence base by offering nuanced insights into women's lived experiences, cultural interpretations of mental health symptoms, and barriers to seeking care (20, 33, 42). These perspectives are crucial for ensuring that screening tools and interventions resonate with local contexts and are acceptable to the women they are intended to serve.

Overall, the findings underscore that maternal mental health in African settings is shaped by an intersection of demographic, socioeconomic, and clinical factors. The diversity of methodologies used contributes to a more comprehensive understanding but also reveals gaps, particularly the need for more longitudinal and intervention studies, as well as more locally validated and culturally sensitive screening strategies. The review demonstrates that depression and anxiety are the most commonly screened maternal mental health conditions across African settings, with prevalence rates varying significantly by tool, population, and cutoff scores used.

## 5 Implications for practice, research and policy

These studies highlight the need for mainstreaming maternal mental health and integrating screening at antenatal and postnatal care points in routine maternal, newborn, and child health (MNCH) programs across Africa. The consistent use of tools such as the EPD and, PHQ-9, will enhance early identification of depressive symptoms and psychological distress among pregnant and postpartum women even in low-resource settings. Integrating mental health into existing maternal and child health platforms offers an opportunity to improve outcomes for mothers and their children while advancing progress toward universal health coverage and the Sustainable Development Goals. Future research should explore the trajectory of maternal mental disorders and their intergenerational impacts and the cultural validation of the maternal mental health screening tools as well as explore intervention models that link screening with effective treatment and support services.

## 6 Limitations of the study

The studies varied widely in methodology, including cross-sectional surveys, cohort studies, randomized controlled trials, qualitative studies, and validation studies, making direct comparison across studies challenging. There was uneven distribution of the studies across Africa, and this may limit transferability of findings.

## 7 Conclusion

This systematic review demonstrates a high burden of maternal mental health problems in Africa, particularly depression and anxiety. These are influenced by a complex interplay of socioeconomic, health, and relational factors and are associated with adverse outcomes for both mothers and their babies. Screening tools such as the EPDS, PHQ-9, SRQ-20, and HSCL-25 were found to be reliable and valid in African populations, although some required contextual adaptation to enhance sensitivity and specificity. However, systemic barriers such as workforce shortages, stigma, and weak referral mechanisms continue to hinder the integration of maternal mental health services into routine care.

The findings therefore highlight the urgent need for maternal mental health to be prioritized and integrate culturally adapted screening within reproductive, maternal, and child health programs in Africa, with the need for health system strengthening, capacity building of health care providers and addressing determinants that contribute to maternal mental health disorders.

## Data Availability

The original contributions presented in the study are included in the article/Supplementary Material, further inquiries can be directed to the corresponding author.
