# Correction: Screening tools for maternal mental health in Africa: a systematic review

**DOI:** 10.3389/fgwh.2026.1920751

**Published:** 2026-08-24

**Authors:** Joyce Jebet, Irene Mageto, Benard Mutwiri, Abednego Ongeso, Ruth Wagathu, Rose Maina, Denis Munene, Rosemary Kithuci, Peter Gatiti

**Affiliations:** 1 School of Nursing and Midwifery, Aga Khan University, Nairobi, Kenya; 2 Library Department, Aga Khan University, Nairobi, Kenya

**Keywords:** maternal mental health, postpartum, pregnancy, screening tools, systematic review

Table error

The two tables have been erroneously interchanged in the published version. Table 3 should be table 2, and table 2 should be table 3.

Incorrect funding

Funder name incorrect

An incorrect Funding statement was provided. The correct funder is Aga Khan University, School of Nursing and Midwifery, Dean's fund. The correct funding statement reads:

The dissemination and publication of the study findings was supported by the Aga Khan University, School of Nursing and Midwifery, Dean’s Fund.

The original version of this article has been updated.

