# Supplementary material for: Screening tools for maternal mental health in Africa: a systematic review
Source: Front Glob Womens Health. 2026 Jun 25;7:1763729. doi: 10.3389/fgwh.2026.1763729 (PMC13346079; doi:10.3389/fgwh.2026.1763729)
Supplement: Supplementary file 1 [file Datasheet1.pdf]

SCREENING TOOLS FOR MATERNAL MENTAL HEALTH IN AFRICA: A SYSTEMATIC REVIEW

Search strategy

Database	Search query	Limits	Results
PubMed	(("screening tools" OR "assessment tools" OR "instrument" OR "screening") AND (((maternal mental health AND ((y_10[Filter]) AND (english[Filter]))) OR (perinatal mental health AND ((y_10[Filter]) AND (english[Filter])))) OR (perinatal mental illness AND ((y_10[Filter]) AND (english[Filter]))) AND ((y_10[Filter]) AND (english[Filter])))) AND ("Africa"[Mesh])	*Year 2014-2024 Language; English*	136
CINAHL	("screening tool*" OR "assessment tool*" OR instrument* OR screening) AND ("maternal mental health" OR "perinatal mental health" OR "perinatal mental illness") AND (MH "Africa+" OR Africa*)	*Year 2014-2024 Language; English*	18
SCOPUS	(TITLE-ABS-KEY (maternal AND mental AND health) OR TITLE-ABS-KEY (perinatal AND mental AND health) OR TITLE-ABS-KEY (perinatal AND mental AND illness) AND TITLE-ABS-KEY ("screening tools" OR "assessment tools" OR "instrument") AND TITLE-ABS-KEY (africa)) AND PUBYEAR > 2013 AND PUBYEAR < 2025 AND (LIMIT-TO (DOCTYPE , "ar") OR LIMIT-TO (DOCTYPE , "re")) AND (LIMIT-TO (LANGUAGE , "English"))	*Year 2014-2024 Language; English*	20
AJOL	maternal mental health AND Screening AND Africa	*Year 2014-2024 Language; English*	79
CORE	maternal mental health AND Screening AND Africa	*Year 2014-2024 Language; English*	234
